# Erroneous Activated Coagulation Time During Atrial Flutter Ablation

**DOI:** 10.1155/2020/3842051

**Published:** 2020-01-06

**Authors:** Sheldon Goldstein, Jazmin Juarez, Agathe Streiff

**Affiliations:** ^1^Associate Professor of Anesthesiology, Montefiore Medical Center/Albert Einstein College of Medicine, 111 East 210^th^ St. 4^th^ Floor Silver Zone, Bronx, NY 10467, USA; ^2^Anesthesiology-Johns Hopkins Medical Center, 1630 Whetstone Way # 622, Baltimore, MD 21230, USA; ^3^Assistant Professor of Anesthesiology, Montefiore Medical Center/Albert Einstein College of Medicine, 111 East 210^th^ St. 4^th^ Floor Silver Zone, Bronx, NY 10467, USA

## Abstract

When performing left-sided catheter ablation, anticoagulation is used to prevent formation of thrombi that might embolize. After heparin administration, appropriate anticoagulation is confirmed by measuring Activated Coagulation Time (ACT). We report a case during which ACT results were erroneous, and review alternatives to the ACT under such circumstances.

## 1. Introduction

Heparin is routinely administered to achieve anticoagulation while performing catheter ablation on the left side of the heart. Anticoagulation is necessary to prevent formation of thrombi on catheters, as thrombi could potentially embolize to coronary, cerebral or peripheral arteries, resulting in myocardial infarction, stroke, or distal vascular occlusion. Anticoagulation is usually monitored with the Activated Coagulation Time (ACT). We report a case of atrial flutter ablation during which multiple ACTs provided erroneous results.

## 2. Case Report

The patient provided written informed consent to report this case.

A 57-year-old female 60 kg, 142 cm, with a past medical history of hypertension, hyperlipidemia, heart failure with preserved ejection fraction and rheumatic mitral valve disease presented for catheter ablation of the left atrium. Fourteen years prior she underwent bio-prosthetic mitral valve replacement and MAZE procedure for atrial fibrillation, after which she was in normal sinus rhythm. This was complicated by valve failure and, within one year, she underwent redo mitral valve replacement with a mechanical prosthesis. Subsequently, she developed atrial fibrillation and underwent posterior pulmonary vein and posterior wall isolation. Six months prior to the current case, she began to experience worsening palpitations. She was found to be in persistent atypical atrial flutter, and it was difficult to control the rate pharmacologically. She was referred to our center for left atrial ablation.

Daily medications included losartan 100 mg, Toprol XL 200 mg, simvastatin 20 mg, and warfarin 2.5 mg. Hemoglobin was 13.7 g/dl and platelet count 262,000/*µ*L. Basic metabolic profile was within normal limits. ProThrombin time/international normalized ratio (PT/INR) were 24.8 seconds (11.8–14.8) and 2.5, respectively. Activated Partial Thromboplastin Time (APTT) was not performed that day, but was 28.5 sec (26.1–33.8) in the remote past.

General anesthesia with endotracheal intubation, and insertion of a postinduction arterial line, were uneventful. Baseline ACT, measured with the i-STAT MN: 300 W Handheld Blood Analyzer (Abbott Laboratories, Abbott Park, Illinois), was 158 seconds. After administration of 167 units/kg of heparin, the next ACT, drawn from a femoral venous sheath after removal of 2-1/2 times the dead space, was 257 seconds. An additional 150 units/kg of heparin was administered. In patients such as this, in whom warfarin is continued and the INR is ≥2.0, a heparin dose half our first bolus often results in an ACT >350 seconds [1]. Still, the ACT of 999 seconds seemed excessive, especially as the first postheparin ACT suggested heparin resistance. Therefore, the ACT was repeated with a new blood sample. Results were 999 seconds on two devices which, although statistically unlikely, raised concern for an error in heparin dosing. Heparin vials were examined and found to be the 1,000 U/ml concentration used in our practice. Intravenous solutions were checked, and heparin was not being administered in error.

ACT of 999 seconds also raised concern for excessive anticoagulation that might result in bleeding. Examination of IV and arterial catheters, and the oropharynx, revealed no bleeding. Two ACTs on a third blood sample, and another on an arterial (fourth) sample reported 999 seconds on three iSTAT devices. Of note, two of these ACTs were performed on cartridges from a second lot. ACT values were now judged unreliable, and cardiologists were concerned for the possibility of inadequate anticoagulation, which might result in thrombosis on the mechanical valve. To confirm adequate anticoagulation APTT, anti-Xa assay for heparin level, and PT/INR were drawn. An ACT was then performed with a cartridge from a newly opened box of cartridges (a third lot), which reported 428 seconds. The APTT returned “quantity insufficient”, even though the blue-top tube had been filled completely. PT/INR returned = 100.7/9.3, confirming anticoagulation was present. Subsequent ACTs, from the newly opened box of cartridges, were within the expected range for the heparin doses administered. Radiofrequency ablation was used to isolate multiple left atrial flutter lines, including two mitral isthmus lines and a roof line. The SVC was isolated as well. After protamine 1.2 mg/kg, ACT decreased to 153 seconds. The patient awoke and was extubated uneventfully. She did well postoperatively, and was discharged the following day. On post-op day one, anti-Xa assay reported unfractionated heparin level >2.28 units/ml. A wide-range heparin assay was requested, and a heparin level of 6.42 units/ml was reported.

## 3. Discussion

During surgery or procedures that require heparin, the ACT has become the test of choice to monitor anticoagulation. This is due to its ease of use, availability at the point-of-care (POC), and rapid time to results, even in patients treated with high-dose heparin. Guidelines for ACT use during cardiac surgery have recently been published [2], but no guidelines exist regarding anticoagulation for electrophysiology procedures. Still, a balance needs to be achieved, and most practitioners maintain the ACT 300–400 seconds during left-sided ablation to minimize risk of thrombi forming on catheters that might embolize to coronary, cerebral or peripheral arteries [3]. Indeed, even with appropriate anticoagulation, thromboembolic complications have been reported in 0.5–4% of procedures [3]. Still, higher ACTs should be avoided, as excessive anticoagulation might worsen bleeding complications such as groin hematoma, retroperitoneal bleeding, and pericardial effusion. It might also lead to pericardial tamponade, and the need for transfusion, pericardiocentesis and/or surgical intervention.

ACT devices are available from several manufacturers. Activators used include celite, kaolin and glass. Results are reported when activated thrombin converts fibrinogen substrate to fibrin. Some ACT devices measure clot formation with mechanical or optical detection. In contrast, in the iSTAT ACT, the substrate is H-D-phenylalanyl-pipecolyl-arginine-p-amino-p-methoxydiphenylamine. When the amide bond at the carboxy- terminus of the arginine residue is cleaved by thrombin, an electro-active compound, NH_3_^+^–C_6_H_4_–NH–C_6_H_4_–OCH is released, and its amperometric detection is reported in seconds [4]. Results are calibrated to match results of the Hemochron (International Technidyne, Edison, NJ) Celite FTCA510 using prewarmed tubes, although calibration can be modified by the user for nonprewarmed tubes.

Causes of prolonged ACT are listed in Table 1. We drew multiple samples from nonheparin containing lines, and drew sufficient volume of dead space to prevent hemodilution. If another cause of prolonged ACT was present, it would have been expected to prolong the baseline value, but that was 158 seconds. Furthermore, the mild rise in ACT from 158 to 257 seconds after 167 U/kg of heparin suggested that the subsequent, much larger increase from 257 to 999 seconds after an additional 150 U/kg was likely to be erroneous.

The i-STAT ACT reports a maximum value of 1,000 seconds, above which >1,000 is reported. Although 999 is a reportable result, the likelihood of measuring multiple ACTs of 999 seconds is exceedingly small. The i-STAT handheld reports multiple numeric error codes, some of which indicate improper cartridge insertion, incorrect volume of test sample, analyzer error, cartridge error, poor contact, and lot expired. However, 999 was not preceded by the word error, and 999 is not even a known error code. Furthermore, Abbott Laboratories indicated they have had no prior report of multiple ACT results of 999 seconds.

After removal from refrigeration, i-STAT cartridges can be stored at 18–30 degrees Celsius, and humidity less than 90%, for two weeks. At our center, when removed from refrigeration, the expiration date was written on each cartridge package. We cannot know the exact date the cartridges were removed from refrigeration, and it is possible an incorrect expiration date was written. Still, it seems unlikely all would report 999 seconds, even if they had been at room temperature for more than two weeks, especially from two different lots. A possible explanation could be that atmospheric conditions were extreme. However, electronic monitoring at the storage site was well within recommended temperature and humidity ranges for 18 days prior to this case. Interestingly, cartridges from both lots were subsequently tested by Abbott Labs, and reported to function normally.

If ACTs are unreliable, APTT is usually the fastest way to confirm heparin-induced anticoagulation even though, after high-dose heparin, the APTT might not clot. Circulating heparin can also be detected with protamine titration performed on the Hepcon (Medtronic-Hemotec, Minneapolis, MN) or Hemochron Response Rx/Dx System (International Technidyne, Edison, NJ). Another option would be tests that assess coagulation in the absence and presence of heparinase. These include the thromboelastogram (Haemonetics Corp., Braintree, MA), the rotational thromboelastometer (Instrumentation Laboratory, Bedford, MA), and the Quantra (Hemosonics, Charlottesville, VA). These options might provide results more rapidly than APTT, depending on their proximity to the patient's location. We sent an anti-Xa assay because, with very high ACT values and a mechanical valve, we wanted the most accurate test to assess heparin effect, and anti-Xa assay is the gold standard. Tests that can confirm the presence of circulating heparin differ in ease of use and time to results, and are compared in Table 2.

Although APTT is commonly used to monitor patients on heparin, it does not correlate well with heparin levels [5]. This is due to variability in heparin preparations and interpatient variability in response to heparin, as well as the fact that hundreds of APTT instrument-reagent combinations exist [6]. Therefore, standardization has not been possible [6]. Reliance on APTT monitoring often results in administration of more heparin than necessary, and may result in an increased incidence of bleeding complications than would be seen with anti-Xa monitoring [7]. Conversely, in a patient on extracorporeal membrane oxygenation, the APTT may be prolonged into the therapeutic range due to factor deficiencies, and result in decreased heparin dosing that may lead to thrombosis [8]. The APTT is also affected by acute phase reactants and the lupus anticoagulant, and may be affected by liver disease [9]. In fact, it has been suggested that Anti-Xa assay supplant the APTT in patients on heparin therapy, as it may offer a smoother dose- response curve, with fewer blood draws, and fewer dosage adjustments [10].

The ACT remains the preferred test to assess heparin effect during routine ablation, since it provides rapid results at the POC. However, in complex clinical situations, performance of anti-Xa assay may be wise. In our case, anti-Xa assay returned on postop day one because at the time of this case the assay was only performed during the daytime. However, the assay is now available 24 hours/day, seven days a week at our center. This is consistent with increasing availability in the United States. Specifically, in 1997, the College of American Pathologists (CAP) proficiency testing survey found that over 4,000 hospitals in France offered an anti-Xa assay, compared with just 14% of hospitals in the United States participating in the survey [6]. In contrast, 90% of participating hospitals in the United States did so in 2018 [11]. This increase is due to the development of automated anti-Xa assays in the early 2000s [12], and the superiority of the anti-Xa assay to assess heparin effect as compared with APTT [10, 13]. Specifically, APTT can remain below the therapeutic range when anti-Xa indicates a therapeutic heparin level, especially in patients with thromboembolic disease. Therefore, patients monitored with APTT alone may receive excessive doses of heparin and be at increased risk of bleeding [13]. As with any test new to the practitioner, caution should be exercised when ordering the anti-Xa assay for the first time. It is important to interpret results based on the treatment used, as the therapeutic range will differ for unfractionated heparin and low molecular weight heparin [14]. Care must also be paid to ordering the correct test, as two other tests have similar names. The factor X activity assay is a clotting time–based assay used to diagnose a deficiency of factor X [14] while the chromogenic factor X assay is used to monitor warfarin effect in patients treated with direct thrombin inhibitors [14]. Only the anti-Xa assay is appropriate to assess heparin effect. Finally, it should be appreciated that although anti-Xa results are reported as heparin units/ml, in fact the assay measures heparin effect.

It is important to perform the ACT in a technically proper and consistent manner. First, cartridges/test tubes to which samples are added should be maintained at recommended storage conditions. Second, an adequate volume of blood should be removed prior to obtaining the ACT sample. This prevents dilution or contamination from drugs from influencing the test result. Third, the sample should be rapidly inserted into a test cartridge or tube, depending on device, and the test initiated rapidly as well, because even in the absence of an activator clot formation may begin in the syringe. Therefore, the ACT result from a blood sample drawn several minutes prior to initiation of testing might report an erroneously shortened clotting time. If an ACT is unexpectedly prolonged, recommended steps to perform are listed in Table 3.

## 4. Summary

Physicians, perfusionists, and nurses need to know proper methods to perform an ACT. If results of a properly performed ACT are inconsistent with the clinical picture, the test should be repeated. If a second ACT is inconsistent with the clinical picture, the possibility that the ACT result is erroneous should be considered, and an alternative test performed to confirm heparin effect. The APTT is the test most often performed to assess heparin effect if ACT is not available. Though additional tests described may confirm the presence of heparin in blood more rapidly, anti-Xa assay is the gold standard to assess heparin effect. Practitioners should become more familiar with this test, both because of its accuracy to indicate heparin effect and the fact that it is now commonly available in hospitals in the United States.

## Figures and Tables

**Table 1:** Causes of prolonged activated coagulation time.

Unfractionated heparin
Low molecular weight heparin
Lupus anticoagulant
Hypothermia
Hemodilution
Hypofibrinogenemia
Factor deficiencies
Thrombocytopenia
Aprotinin

**Table 2:** Tests available to confirm presence of heparin in blood.

Test	Platform	Location	Time to result	Ease of performance
ACT	Multiple	POC	<10 mins	Simple

Protamine titration	Hepcon HMS	POC	<10 mins	Simple
	Hemochron Rx/Dx			

*r*-time and Heparinase *r*-time	TEG	POC or central lab	<15 mins	Moderate

INTEM and HEPTEM	ROTEM	POC or central lab	<15 mins	Moderate

CT and HCT	Quantra	POC	<15 mins	Simple

APTT	Multiple	Central lab	45–90 mins	Complex

Anti-Xa	Multiple	Central lab	45–90 mins	Complex

ACT = activated coagulation time; POC = point-of-care; HMS = heparin management system; TEG = thromboelastography; INTEM = screening test of hemostasis performed on ROTEM; HEPTEM = INTEM performed in presence of heparinase; ROTEM = rotational thromboelastometry; CT = clotting time; HCT = heparinase clotting time; APTT = activated partial thromboplastin time; Anti-Xa = anti-Xa assay. *Note:* Time to anti-Xa result assumes a lab that reconstitutes reagents and runs controls daily.

**Table 3:** Recommended steps when ACT is unexpectedly prolonged.

(1) Examine patient for signs of excessive bleeding
(2) Confirm correct doses of heparin administered
(3) Confirm no heparin being administered in error via intravenous lines
(4) Perform ACT in duplicate, with two devices
(5) Repeat ACT with a Cartridge/test tube from a new lot
(6) Send APTT to central laboratory
(7) Consider tests listed in Table 2 to confirm the presence of heparin in blood
(8) Consider Anti-Xa assay, if available
